# Examining hypertension risk among Black and White breast cancer survivors

**DOI:** 10.1002/cam4.6929

**Published:** 2024-01-12

**Authors:** Arnethea L. Sutton, Jian He, Wendy Bottinor, Susan Hong, Kristyn Mitchell, Anika L. Hines

**Affiliations:** ^1^ Department of Kinesiology and Health Sciences Virginia Commonwealth University Richmond Virginia USA; ^2^ VCU Massey Comprehensive Cancer Center Richmond Virginia USA; ^3^ Division of Cardiology, Department of Internal Medicine VCU School of Medicine Richmond Virginia USA; ^4^ Division of Hematology/Oncology, Department of Internal Medicine VCU School of Medicine Richmond Virginia USA; ^5^ VCU School of Medicine Richmond Virginia USA; ^6^ Department of Health Behavior and Policy VCU School of Medicine Richmond Virginia USA

**Keywords:** breast cancer, cancer treatment‐related cardiac dysfunction, cardio‐oncology, geographical disparities, hypertension, racial disparities

## Abstract

**Purpose:**

Breast cancer survivors are at increased risk of cardiovascular dysfunction following their diagnosis; however, hypertension remains underexplored within this context. This retrospective cohort study examined the incidence of hypertension in breast cancer survivors and the association of race with hypertension risk among them.

**Methods:**

Data for this study were abstracted from the electronic health records of women diagnosed with Stages I–III breast cancer. Incident hypertension diagnosis was identified through International Classification of Diseases codes. Bivariate associations were tested using Student's *t*‐test and chi‐squared test of independence. Bivariable Cox regression analysis was used to determine demographic and clinical factors that may have been associated with the development of hypertension.

**Results:**

A total of 664 women were included. Most women were 50 years of age or younger (52.0%), White (33.0% Black), and received a mastectomy (80.6%). Overall, 45.5% of the cohort developed hypertension. The 1‐year hypertension‐free survival estimates were 47% (95% confidence interval [CI], 41–54) in Black women and 73% (95% CI, 69–77) in White women (*p* < 0.0001). Besides race, statistically significant predictors of hypertension included: age greater than 50 (vs. ≤50) (adjusted Hazard Ratio [HR]: 1.40; 95% CI, 1.09–1.80) and residing in a non‐metropolitan area (vs. metropolitan) (adjusted HR: 1.60; 95% CI, 1.19–2.16).

**Conclusions:**

This study suggests that breast cancer survivors who are older, Black, or residing in non‐metropolitan areas may benefit from added surveillance and hypertension prevention strategies during treatment. Future studies are needed to identify contributors to the observed racial and geographic disparities.

## 1 INTRODUCTION

Breast cancer survivors are at increased risk of developing cancer therapy‐related cardiac dysfunction (CTRCD) following treatment with potentially cardiotoxic treatments such as anthracycline‐based chemotherapies (e.g., doxorubicin) and trastuzumab.^1^, ^2^, ^3^, ^4^ Studies examining CTRCD in breast cancer survivors have traditionally focused on left ventricular ejection fraction (LVEF) decline, heart failure, and cardiomyopathy, leaving a knowledge gap in the understanding of the onset of hypertension following a breast cancer diagnosis and receipt of cardiotoxic treatment. One recent cohort study by Kwan et al. reported a higher risk of hypertension in breast cancer survivors than in women without breast cancer (10.9% vs. 8.9%, respectively).^5^ It is salient to understand the manifestation of hypertension following a breast cancer diagnosis, specifically for those who are at increased risk due to therapies and because it may contribute to other forms of cardiovascular disease and events.

Studies have demonstrated a higher risk of CTRCD among women who have hypertension when receiving treatment with potentially cardiotoxic treatment for breast cancer when compared to women who do not have hypertension.^6^, ^7^, ^8^ What has not been fully characterized are the risk factors for hypertension, beyond cardiotoxic treatment. Studies in women without breast cancer have identified risk factors for hypertension to include family history, obesity, and older age.^9^ Studies have also shown that Black women are more likely to develop hypertension when compared to White women.^10^, ^11^, ^12^ In order to further our understanding of hypertension development in breast cancer survivors, we examined the risk of hypertension following a breast cancer diagnosis in Black and White women who received potentially cardiotoxic therapies utilizing hospital data, and we identified demographic and clinical factors that were associated with hypertension risk. We hypothesized that Black women, older women, and women with diabetes would have a higher incidence of hypertension when compared to their counterparts.

## 2 METHODS

### 2.1 Study design and patient selection criteria

This was a retrospective study of electronic health record data from Virginia Commonwealth University Massey Comprehensive Cancer Center, a National Cancer Institute designated cancer center. The primary purpose of the parent study was to assess racial differences in CTRCD (e.g., heart failure) among women receiving cardiotoxic treatments. Women represented in this analysis self‐identified as African American/Black or Caucasian/White and were diagnosed with Stages I–III invasive breast cancer between 2005 and 2019. All women received anthracycline‐based chemotherapy and/or trastuzumab. Women were excluded if they were missing smoking data, had a hypertension diagnosis, or were prescribed antihypertensive medications prior to or upon their breast cancer diagnosis.

### 2.2 Definition of incident hypertension

Incident hypertension following a breast cancer diagnosis was identified via International Classification of Diseases versions 9th or 10th revisions, clinical modification (ICD‐9‐CM; ICD‐10‐CM) diagnosis codes (i.e., 401.xx; I10) in inpatient and outpatient files or at least two systolic blood pressures >140 mmHg and/or diastolic blood pressures >90 mmHg.

### 2.3 Demographic and clinical variables

Demographic data included race, age at cancer diagnosis, insurance status (e.g., private, government‐issued, and uninsured), marital status (i.e., married vs. unmarried), and geographic area of residence at the time of diagnosis ascertained through Rural Urban Continuum Codes (i.e., metropolitan vs. non‐metropolitan). Clinical data included American Joint Committee on Cancer (AJCC) stage, hormone receptor status (e.g., estrogen [ER], progesterone [PR]), surgery type (e.g., lumpectomy, mastectomy), body mass index (BMI), smoking status (e.g., never smoker, former smoker), and diabetes status. All data, abstracted from the electronic health record, were collected for women diagnosed between 2005 and 2019. For each patient, information was collected from the time of diagnosis to death or censoring on December 31, 2020.

### 2.4 Data analysis

A summary of baseline demographic and clinical characteristics stratified by women with or without hypertension was calculated using mean ± standard deviation (SD) for continuous data and frequency (percentage) including missing values for categorical data. Two‐sample unpaired *t*‐test under the equal variance assumption was used to test the mean difference between the women with hypertension and those without hypertension for each continuous variable; chi‐squared test of independence was used to test the proportions between the aforementioned two groups for each categorical variable.

Kaplan–Meier (KM) survival curves for all women and stratified by race (Black and White) were plotted to give a graphical representation of hypertension‐free survival probability following a breast cancer diagnosis. A log‐rank test was used to compare the hypertension‐free survival curves by race. Time‐dependent Cox models with time from a breast cancer diagnosis to development of hypertension, change in smoking status, death or loss to follow‐up, whichever comes first, as the time scale were used to estimate hazard ratios (HR). If there were any change in smoking status for a subject, her time scale would be divided at the time of change. A bivariable model was fitted for each covariate to select statistically significant predictors for inclusion in the multivariable model and adjust for potential confounding factors. The multivariable time‐dependent Cox model was used to compare the instantaneous risk of hypertension following a breast cancer diagnosis between patients based on selected covariates (race, age at diagnosis, marital status, insurance status, geographic location, BMI, smoking status, ER and PR status, surgery type, and whether the patient had diabetes). Age was dichotomized as <50 years and ≥50 years due to median age of survivors at diagnosis, All hypothesis tests were two‐tailed with a *p*‐value <0.05 considered statistically significant. Statistical analyses were performed using R version 4.3.1 with the following packages: tidyverse 2.0.0, survival 3.5.5, survminer 0.4.9, and gtsummary 1.7.1.^13^

## 3 RESULTS

### 3.1 Patient characteristics

A total of 664 women were included in this analysis. The mean age of the cohort was 50.31 years old (SD = 10.7) (Table 1). A majority of the cohort were 50 years of age or younger (52.0%), White (67.0%), resided in metropolitan areas (85.4%), received anthracycline therapy (77.3%), never smoked cigarettes (63.3%), and did not have diabetes (94.9%).

**TABLE 1:** Baseline demographic and clinical characteristics of breast cancer survivors stratified by hypertension status.

Variables	Overall (*N* = 664)	Hypertension (*N* = 302)	No hypertension (*N* = 362)	*p*‐value
Age (mean ± SD)	50.3 ± 10.7	52.0 ± 10.7	48.8 ± 10.5	0.0002
Age at diagnosis, *n* (%)				0.003
≤50	345 (52.0)	138 (45.7)	207 (57.2)	
>50	319 (48.0)	164 (54.3)	155 (42.8)	
Race, *n* (%)				<0.0001
Black	219 (33.0)	140 (46.4)	79 (21.8)	
White	445 (67.0)	162 (53.6)	283 (78.2)	
Marital status, *n* (%)				<0.0001
Married	385 (58.0)	144 (47.7)	241 (66.6)	
Unmarried	279 (42.0)	158 (52.3)	121 (33.4)	
Insurance status, *n* (%)				0.0005
Private	423 (63.7)	169 (56.0)	254 (70.2)	
Government issued	114 (17.2)	70 (23.2)	44 (12.2)	
Not insured	62 (9.3)	32 (10.6)	30 (8.3)	
Self‐pay	61 (9.2)	28 (9.3)	33 (9.1)	
Missing	4 (0.6)	3 (1.0)	1 (0.3)	
Geography, *n* (%)				0.0003
Non‐metro	94 (14.2)	59 (19.5)	35 (9.7)	
Metro	567 (85.4)	241 (79.8)	326 (90.1)	
Missing	3 (0.5)	2 (0.7)	1 (0.3)	
BMI, *n* (%)				<0.0001
Normal/underweight	195 (29.4)	65 (21.5)	130 (35.9)	
Obesity	255 (38.4)	150 (49.7)	105 (29.0)	
Overweight	214 (32.2)	87 (28.8)	127 (35.1)	
Surgery type				0.004
Lumpectomy or excisional biopsy	84 (12.7)	25 (8.3)	59 (16.3)	
Mastectomy	535 (80.6)	257 (85.1)	278 (76.8)	
Other	29 (4.4)	10 (3.3)	19 (5.2)	
Missing	16 (2.4)	10 (3.3)	6 (1.7)	
Anthracycline chemotherapy				0.619
No	151 (22.7)	66 (21.9)	85 (23.5)	
Yes	513 (77.3)	236 (78.1)	277 (76.5)	
Trastuzumab				0.909
No	425 (64.0)	194 (64.2)	231 (63.8)	
Yes	239 (36.0)	108 (35.8)	131 (36.2)	
Radiation				0.88
No	180 (27.1)	81 (26.8)	99 (27.3)	
Yes	484 (72.9)	221 (73.2)	263 (72.7)	
Smoking history				0.006
Current smoker	92 (13.9)	55 (18.2)	37 (10.2)	
Former smoker	152 (22.9)	72 (23.8)	80 (22.1)	
Never smoker	420 (63.3)	175 (57.9)	245 (7.7)	
Diabetes				<0.0001
No	630 (94.9)	275 (91.1)	355 (98.1)	
Yes	34 (5.1)	27 (8.9)	7 (1.9)	

### 3.2 Incidence of hypertension

We identified 302 women with hypertension following breast cancer (45.5%). In bivariate analysis, 64% of Black survivors developed hypertension compared to 36% of White survivors (*p* < 0.0001). Among survivors who were married, 37% developed hypertension while 57% of survivors who were unmarried developed hypertension (*p* < 0.0001). Residential geography (*p* = 0.0003), insurance status (*p* = 0.0005), BMI (*p* < 0.0001), smoking status (*p* = 0.006), and diabetes (*p* < 0.0001) were associated with hypertension.

**FIGURE 1:**
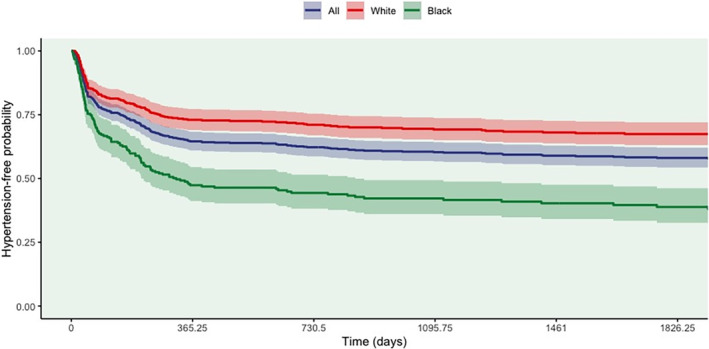
Hypertension‐free survival for all women, Black women only, and White women only.

Figure 1 depicts Kaplan–Meier curves for hypertension‐free survival for all women and stratified by race. Among Black survivors, the 1‐year hypertension‐free survival estimate was 47% (95% confidence interval [CI], 41–54) compared to 73% (95% CI, 69–77) in White survivors (*p* < 0.0001). The median hypertension free survival estimates were 10 years for all survivors and 316 days for Black women. The median hypertension‐free survival for White survivors could not be estimated, since their hypertension‐free survival probability never fell below 0.50 during the study period.

After adjusting for the following statistically significant covariates and the confounding factors, i.e., marital status, smoking status, ER and PR status, as well BMI and diabetes, we came to the following conclusions: Black survivors were more likely to develop hypertension when compared to White survivors (HR, 1.84; 95% CI, 1.39–2.43) (Table 2). Women greater than 50 years of age had a substantially higher risk of developing hypertension than women 50 years of age or younger (HR, 1.40; 95% CI, 1.09–1.80) as did women who had government‐issued insurance (vs. private insurance) and those living in non‐metropolitan areas (vs. metropolitan areas) (HR: 1.55; 95 CI, 1.13–2.12 and HR: 1.60; 95% CI, 1.19–2.16, respectively). Survivors with normal/underweight (HR: 0.58; 95% CI, 0.42–0.81) and those with overweight (HR: 0.68; 95% CI, 0.51–0.90) were less likely to develop hypertension when compared to survivors with obesity. Hypertension risk was lower for survivors who underwent lumpectomy (vs. mastectomy) (HR, 0.54; 95% CI, 0.36–0.83). Lastly, women who had diabetes had a substantially higher risk of developing hypertension than survivors who did not (HR, 2.14; 95% CI, 1.34–3.42).

**TABLE 2:** The time‐dependent Cox model for association between  characteristics and incident hypertension.

Baseline characteristic	Hazard ratio	95% CI
Race		
Black	1.84	1.39–2.43***
White	1.00	Reference
Age at diagnosis		
>50	1.40	1.09–1.80**
≤50	1.00	Reference
Marital status		
Unmarried	1.15	0.89–1.49
Married	1.00	Reference
Insurance status		
Government‐issued	1.55	1.13–2.12**
Not insured	1.06	0.71–1.59
Self‐pay	0.86	0.56–1.31
Private	1.00	Reference
Geography		
Non‐metropolitan	1.60	1.19–2.16**
Metropolitan	1.00	Reference
Body mass index		
Normal/underweight	0.58	0.42–0.81**
Overweight	0.68	0.51–0.90**
Obesity	1.00	Reference
Estrogen receptor status		
Positive	0.90	0.60–1.33
Negative	1.00	Reference
Progesterone receptor status		
Positive	0.94	0.64–1.39
Negative	1.00	Reference
Surgery type		
Lumpectomy or excisional biopsy	0.54	0.36–0.83**
Other	0.59	0.31–1.11
Mastectomy	1.00	Reference
Smoking status		
Current smoker	1.34	0.96–1.88
Former smoker	0.89	0.66–1.19
Never smoker	1.00	Reference
Diabetes		
Yes	2.14	1.34–3.42**
No	1.00	Reference

** *p* < 0.01.

*** *p* < 0.001.

## 4 DISCUSSION

Our study demonstrated that Black women diagnosed with breast cancer who receive anthracyclines and/or trastuzumab were more likely to develop hypertension compared to White women treated with these agents. The incidence of hypertension was 217.68 per 1000 women‐years among Black women compared with 69.45 per 1000 women‐years among White women. Additional demographic and clinical factors, such as older age, obesity, and diabetes, well‐founded risk factors for hypertension,^14^ were associated with higher risk of hypertension in our study. The aforementioned factors are well‐founded risk factors for hypertension. Living in a non‐metropolitan area (vs. a metropolitan area) was associated with increased risk of hypertension. The disproportionately increased risk for hypertension among Black cancer survivors may contribute to the well described disparities in cardiovascular outcomes after cancer diagnosis.^15^, ^16^

Similar to previous studies, we found that after controlling for traditional hypertension risk factors, Black breast cancer survivors had a higher prevalence of hypertension than White survivors at the time of their breast cancer diagnosis.^17^, ^18^ Our study further contributes to the literature by examining the rates of hypertension development in previously normotensive women after a breast cancer diagnosis. Reasons for the observed disparity in our sample may be due to non‐clinical or non‐traditional risk factors, such as those associated with the lived experiences of Black women.

Further investigation to understand the underlying factors that contribute to this disparity is needed.^19^ Among non‐cancer population, studies on racial disparities in hypertension among individuals without cancer are plentiful and provide a template for further investigation into this finding among breast cancer survivors. For example, the associations between experiences with racial discrimination and hypertension risk, management, and outcomes among Black people have been documented on the individual^20^, ^21^, ^22^, ^23^ and structural levels.^24^, ^25^, ^26^ Additional work is needed to understand what multilevel factors (e.g., psychosocial, care delivery) contribute to higher rates of hypertension among Black women and its escalated development following a breast cancer diagnosis relative to White women. This may include investigating the downstream impact of social drivers of health on cancer care and on the lived experiences (e.g., psychosocial stress) of Black women following a breast cancer diagnosis.

Women who resided in non‐metropolitan areas when diagnosed had a higher risk of hypertension when compared to women living in metropolitan areas. While these findings have not been previously reported in breast cancer survivors, studies in individuals with other cancer types and in individuals without cancer have been mixed. A recent study of individuals with head and neck cancer reported a lower prevalence of hypertension among individuals living in rural areas compared to those living in urban areas.^27^ Conversely, in a study assessing pre‐pregnant hypertension, women living in rural areas had a higher prevalence of hypertension than women living in urban areas. Women living in rural areas may have presented at diagnosis with higher blood pressures, due, in part, to lack of resources and barriers to care often experienced by rural residents. Additionally, behaviors associated with hypertension including smoking and physical inactivity are more prevalent in rural residents, particularly for cancer survivors.^28^, ^29^, ^30^, ^31^ Future research identifying behavioral and structural factors contributing to hypertension in women living in rural areas is needed.

In our study, women who underwent a mastectomy were at increased risk of developing hypertension when compared to women who underwent lumpectomy. To our knowledge, this finding has not been reported elsewhere. In many cases, women who undergo a mastectomy are more likely to have more advanced disease, which may also correlate to the more aggressive potentially cardiotoxic anthracycline‐based chemotherapy. However, in this study anthracycline use (vs. no anthracycline use) was not associated with increased hypertension risk. A recent study by the National Cancer Institute, found that women who undergo mastectomy (vs. lumpectomy) reported more physical symptoms (e.g., numbness, lymphedema) as a result of their surgery.^32^ Hypertension may be a short‐ or long‐term sequela of physical symptoms associated with a mastectomy.

There are notable strengths in this study. This cohort is composed of women who all received cardiotoxic treatment; this is important because it has been well established that anthracyclines and trastuzumab contribute to CTRCD; therefore, future work must understand additional factors among those who receive these treatments. Data for this study were abstracted from the EHR from a safety‐net health system, allowing us to understand hypertension risk among a demographically and economically diverse group of survivors. There are also limitations to note. EHR data are limited in that they do not allow for the investigation of the roles of non‐clinical factors that may contribute to hypertension onset and disparities, therein. Also, we did not include information on exposure to adjuvant hormone therapy or exposure to cisplatin, both of which may contribute to hypertension risk.

## 5 CONCLUSIONS

Our results demonstrated a significantly increased risk for hypertension within a year of breast cancer diagnosis among Black survivors compared with White survivors. This may contribute to the overall disparity in cardiovascular outcomes among survivors from different racial groups. Further studies should be performed to determine if enhanced surveillance and treatment of hypertension among breast cancer survivors who are Black, over 50 years of age, or reside in a non‐metropolitan setting may improve cardiovascular outcomes.

## AUTHOR CONTRIBUTIONS

**Arnethea L. Sutton:** Conceptualization (lead); data curation (equal); funding acquisition (lead); investigation (equal); methodology (equal); project administration (equal); resources (lead); supervision (lead); writing – original draft (equal); writing – review and editing (equal). **Jian He:** Formal analysis (lead); methodology (equal); writing – original draft (supporting); writing – review and editing (supporting). **Wendy Bottinor:** Writing – original draft (supporting); writing – review and editing (supporting). **Susan Hong:** Conceptualization (supporting); writing – original draft (supporting); writing – review and editing (supporting). **Kristyn Mitchell:** Data curation (supporting); writing – original draft (supporting); writing – review and editing (supporting). **Anika L. Hines:** Conceptualization (supporting); supervision (supporting); writing – original draft (supporting); writing – review and editing (supporting).

## FUNDING INFORMATION

This research was funded by the National Cancer Institute K99CA256038 (Sutton). It was also supported, in part, by the National Heart Lung and Blood Institute (K01JL152011), the American Heart Association (19CDA34760181 and 940,494), and the To‐morrow's Research Fund St. Baldrick's Scholar Award (636214). Services in support of this study were generated by the VCU Massey Cancer Center Biostatistics Shared Resources and the Bioinformatics Core, supported with funding from the NIH‐NCI Cancer Center Support Grant P30 CA016059 and through REDCap provided by the Clinical and Translational Sciences Award from the National Center for Advancing Translational Sciences (No. UL1TR002649) and KL2TR002648.

## CONFLICT OF INTEREST STATEMENT

The authors declare that they have no conflict of interest.

## ETHICS STATEMENT

All procedures performed in studies involving human participants were in accordance with the ethical standards of the institutional and/or national research committee and with the 1964 Helsinki Declaration and its later amendments or comparable ethical standards. The study was approved by the Institutional Review Board (IRB) at Virginia Commonwealth University. A waiver of consent/exempt was granted by the IRB.

## Data Availability

Availability of data and material. Data archiving is not mandated but will be made available on reasonable request.
